# Biomedical Applications of Biomolecules Isolated from Methanotrophic Bacteria in Wastewater Treatment Systems

**DOI:** 10.3390/biom11081217

**Published:** 2021-08-16

**Authors:** Rana Salem, Ahmed ElDyasti, Gerald F. Audette

**Affiliations:** 1 Department of Chemistry, York University, Toronto, ON M3J 1P3, Canada; rsalem@yorku.ca; 2 Department of Civil Engineering, York University, Toronto, ON M3J 1P3, Canada; ahmed.eldyasti@lassonde.yorku.ca; 3 The Centre for Research on Biomolecular Interactions, York University, 4700 Keele Street, Toronto, ON M3J 1P3, Canada

**Keywords:** methanotrophs, methanobactin, ectoine, biogas, S-layer, polyhydroxyalkonate (PHA), polyhydroxybutyrate (PHB), methane monooxygenase (MMO), exopolysaccharide (EPS), single cell protein (SCP), wastewater treatment, biomedical applications

## Abstract

Wastewater treatment plants and other remediation facilities serve important roles, both in public health, but also as dynamic research platforms for acquiring useful resources and biomolecules for various applications. An example of this is methanotrophic bacteria within anaerobic digestion processes in wastewater treatment plants. These bacteria are an important microbial source of many products including ectoine, polyhydroxyalkanoates, and methanobactins, which are invaluable to the fields of biotechnology and biomedicine. Here we provide an overview of the methanotrophs’ unique metabolism and the biochemical pathways involved in biomolecule formation. We also discuss the potential biomedical applications of these biomolecules through creation of beneficial biocompatible products including vaccines, prosthetics, electronic devices, drug carriers, and heart stents. We highlight the links between molecular biology, public health, and environmental science in the advancement of biomedical research and industrial applications using methanotrophic bacteria in wastewater treatment systems.

## 1. Introduction

In recent years, a global movement has engaged targeting the development of alternative bio-based therapeutic products for biomedical applications in order to reduce or eliminate the adverse side effects associated with the use of non-biocompatible compounds by the human immune system [1,2]. A broad spectrum of naturally occurring compounds derived from animals, plants, or microbes has been tested for their employment in modern medicine [3]. In the early 2000s, twenty naturally derived therapeutic drugs were developed and brought to market [4], though this clearly was not the first instance of using biologically-derived molecules in medicine. The use of naturally occurring compounds has been documented in ancient civilizations such as Egypt and China, where they relied on plant extracts and honey for remediation and healing purposes [5], and of course Alexander Fleming’s discovery of penicillin opened the door for the use of antibiotics to fight infection, but also highlighted the potential for microbially synthesized products in the pharmaceutical and medical industries [6]. The employment of bioactive composites for use in many industries has become a more robust and efficient process in many applications including: (i) the manufacturing of biopolymers, nanoparticles, pigments for the productions of drug capsules, optical fibers, electronic devices, and paint [7,8,9], (ii) the extraction of catalytic enzymes, organic acids, and surfactants to employ in drug, food, and soap industries [10,11,12], and (iii) the use of organic vitamins, lipids, and proteins, and microbial metabolites to generate synthetic hormones, nutritional supplements, targeted therapies, vaccines anti-cancer agents, anti-inflammatory drugs, and the overall medicinal industry [13,14,15,16,17]. Interestingly, wastewater treatment plants (WWTPs) comprise an important asset for its public health, environmental and economic contributions, in addition to their function as a remediation facility. WWTPs encompass an important revenue stream for their role in resource recovery [18]. More recently, WWTPs have been viewed as biorefinery facilities; they exploit the organic matter within wastewater as microbial substrates in order to sustainably generate electricity, remove contaminants, and recover resources [19]. WWTPs can be considered large biodiverse microbial populations that are distinct within each stage of the water treatment process. Each microbial population can also be characterized with the ability to produce a variety of value-added products, each of which is suitable for use in a variety of implementations in different sectors of biomedical industries [18,20]. WWTPs have a broad variety of different important bacterial genera such as purple sulfur bacteria, ammonia oxidizing bacteria, nitrate oxidizing bacteria, *Pseudomonas*, *Mycobacterium*, and *Methylobacterium* [21]. Each bacterial population represents important contributors for resource recovery for the production of biopolymers, catalytic enzymes, lipids, and proteins [19]. While bioproduct resource recovery from WWTPs can be more challenging compared to purely synthetic methods, it is a more sustainable option and is essential to overcome limitations in resource availability. For instance, more synthetic routes for production of high-value biomolecules require cost-intensive processes and bio-refineries for realistic applications. On the other hand, while there are many technical challenges in the processes of optimal bacterial cultivation, biomolecule extraction, and purification from resource pools such as WWTPs, many of these challenges are offset by the large and renewable feedstock, leading to lower input costs associated with bioproduct development. In this review we highlight the role of methane oxidizing bacteria, namely the methanotrophs found in anerobic digestion processes of WWTPs, as a multiple high-value bioproduct generating system with diverse potential biomedical applications. Furthermore, we provide an overview of the enzymatic pathways employed by methanotrophs to generate different metabolites and demonstrate the dynamic interactions of different types of biomolecules.

## 2. Methanotrophic Bacteria in Water Treatment Systems

Methanotrophic bacteria have a unique metabolism that relies on a single carbon substrate, methane. However, its sophistication allows it to manufacture a mixture of value-added organic compounds. Methanotrophs utilize methane gas as an electron donor in order to produce sufficient energy required for cellular growth and biosynthesis of different metabolites [22]. While methanotrophs might not be a top producer for certain products, this is compensated by its ability to perform multiple roles simultaneously which begins with the oxidation of methane gas to produce methanol, an important biofuel [23]. Methane gas is the second most potent greenhouse gas after carbon dioxide; therefore, methanotrophic bacteria’s ability to mitigate this harmful biogas is crucial in the global methane cycle. WWTPs are responsible for an estimated 4% of the global methane production; the gas is normally flared into the atmosphere contributing to global warming [24]. Whenever methanotrophs are present in WWTPs, they are associated with the availability of methane gas produced from the anaerobic digestion process, and with the right allocations the generated biogas can be exploited for resource recovery purposes [25].

Methanotrophs are Gram-negative bacteria and are a subgroup of a broader bacterial group known as methylotrophs [26]. They are distinct in their reliance on methane oxidization, unlike methylotrophs that have the potential to utilize different single carbon substrate such as methanol, halomethanes, and methylated amines [27]. Thus, aerobic methanotrophs metabolism relies on the role of oxygen to oxidize the methane substrate to ultimately generate carbon dioxide (CO_2_) and water [28].

**Figure 1:**
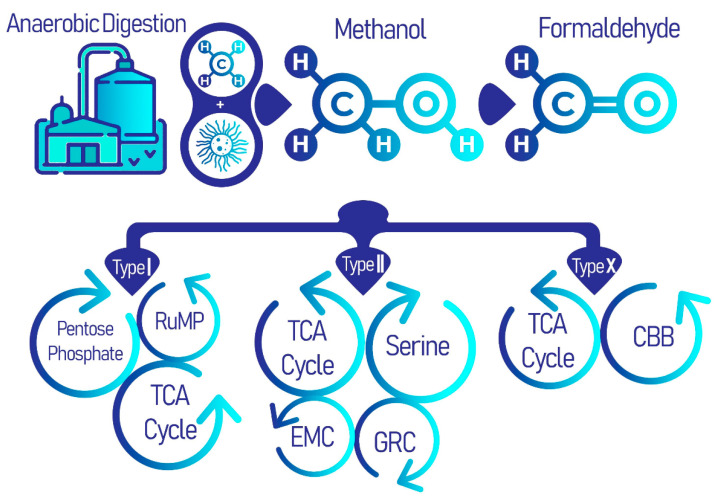
Methanotrophs in anaerobic digestion processes of WWTPs using the common methane oxidation pathway, which branches out for distinct metabolic cycles for each type of methanotrophs: types I, II, and X. It is these distinct secondary metabolic pathways, including the pentose-phosphate pathway (PPP), tricarboxylic acid (TCA) cycle, serine cycle, ethyl malonyl-CoA (EMC) pathway, glyoxylate regeneration cycle (GRC) and the Calvin-Benson-Bassham (CBB) cycle, that produce a diversity of useful biomolecules.

There are three types of aerobic methanotrophs that differ phylogenetically, types I, II, or X, each of which follow a distinct metabolic pathway. All three types share a common methane oxidation pathway to produce formaldehyde, after which, each group carries on in a different enzymatic pathway. Type I methanotrophs employ the ribulose monophosphate (RuMP), and type II methanotrophs utilize the serine pathway (Figure 1) [29]. Type X methanotrophs exhibit similarities with type I methanotrophs in that they use the RuMP pathway; however, it differs from type I as they also have low concentrations of the serine pathway enzyme ribulose–bisphosphate carboxylase [30].

### 2.1. Taxonomy and Phenotype

Aerobic methanotrophs are taxonomically classified based on their phenotype, ability for spore formation, possession of specific membrane bound proteins, and their metabolic properties [24]. There are three main groups of methane oxidizing bacteria, types I, II, and X, each of which undertake a unique enzymatic pathway [31]. Type I and X methanotrophs belong to gamma-proteobacteria and reside in families *Methylococcaceae* and *Methylothermaceae* [32], whereas type II methanotrophs belong to alpha-proteobacteria from families *Methylocystaceae* and *Beijerinckiaceae*. Over time other groups of methanotrophs have emerged, including filamentous methane oxidizers with unusual methane monooxygenase, and extremely acidophilic bacteria of the phylum *Verrucomicrobia*. The classifications of methane-utilizing bacteria has of course evolved; however, the terms types I, II, and X are still commonly used when discussing the methanotrophs due to the distinctiveness of metabolic pathways for carbon fixation across phylogenetic groupings [26,30]. Morphologically, methanotrophs exhibit red coloring under Gram-staining and have various physical morphologies. For instance, type X methanotrophs are mainly found as paired cocci, while type II methanotrophs are crescent shaped rods and can occur in rosettes, and finally, type I methanotrophs can be found as either single cocci or rods [24].

### 2.2. EcoPhysiology

Aerobic methanotrophic bacteria inhabit oxic zones where oxygen is present as an electron acceptor and organic carbon, methane, is present for cellular biosynthesis, such as soils and freshwater, rice paddies, and WWTP sludge [33]. Most methanotrophs are mesophilic and prefer neutral pH; however, some thermophilic genera inhabit areas with high methane profusion and high temperatures such as volcanoes and soil paddies [34,35]. Moreover, a few psychrophilic species found in temperatures between 4 and 10 °C mainly belonging to type I methanotrophs have been reported in arctic regions [36,37,38].

**Figure 2:**
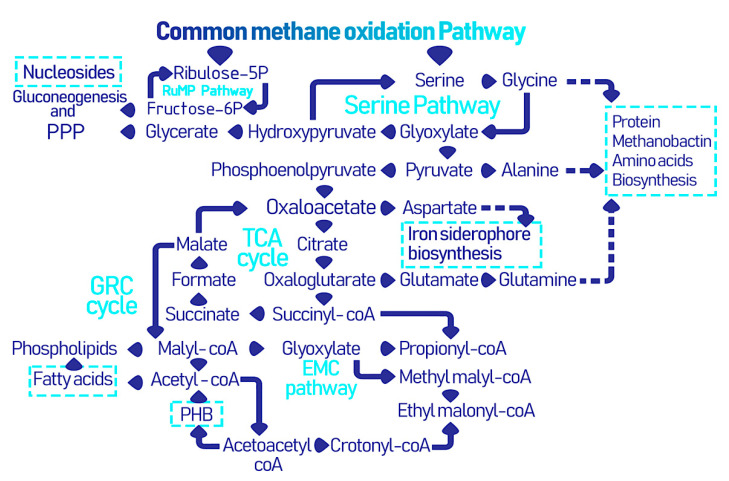
The collective metabolic pathways within methanotrophs leading to the formation of industrially and biomedically valuable biomolecules and biopolymers [25].

Different types of methanotrophs follow distinct metabolic pathways after the oxidation of methane to formaldehyde and formate, which lead to the formation of industrially and biomedically valuable biomolecules and biopolymers as illustrated in Figure 2. First, methane is converted to methanol by the action of methane monooxygenase (MMO), then methanol dehydrogenase (MDH) further oxidizes methanol into formaldehyde [39]. There are two types of MMO: (i) the membrane bound and copper reliant particulate (p)MMO and (ii) soluble (s)MMO. Nonetheless, the *pmoA* gene encoding for pMMO is considered a universal marker for methanotrophic bacteria and is expressed by nearly all types of methanotrophs. Conversely, sMMO is typically expressed by type II and some type X methanotrophs under conditions of copper scarcity, where it utilizes iron as an alternative [40]. Following the common methane oxidation pathway, type I methanotrophs undergo the RuMP cycle, which is responsible for formaldehyde assimilation and detoxification. In the RuMP cycle, formaldehyde is fixed with ribulose 5-phsosphate to form 3-hexulose-6-phophate, which is then converted to glyceraldehyde-3-phosphate to finally generate pyruvate. There is an interplay between the RuMP pathway and the pentose phosphate pathway (PPP), where the latter ensures the regeneration of ribulose-5-phsosphate, while the former produces fructose-6-phosphote that is further metabolized via the PPP [41]. Thereafter, the pentose phosphate shunt is responsible for the generation of NADPH and ribose, which is of course important for the formation of nucleotide based biological molecules. These two pathways are crucial precursors of amino acid, nucleotide, and lipid biosynthesis. Pyruvate is further converted into acetyl-CoA for incorporation into the tricarboxylic acid (TCA) cycle and the electron transport chain for energy generation [27].

In type II methanotrophs, formaldehyde produced from the common metabolic pathway is further oxidized to formate and then converted to 5,10-methylenetetrahydrofolate to form serine and yields phosphoglycerate in several stepwise reactions of the serine cycle. The serine pathway requires 3 ATP and 2 NADH for activation unlike the RuMP cycle which only requires a single ATP. The serine pathway is simultaneously synced with the ethyl malonyl Co-A (EMC) pathway which includes several CoA thioesters, starting with malyl-CoA in the TCA which is converted into acetyl-CoA and eventually ethyl malonyl-CoA is cleaved into glyoxylate and propionyl-CoA where the former is an intermediate for the formation of oxaloacetate and succinyl-CoA for the latter, which are important originators for creating high-end products [42]. Furthermore, an intermediary cycle for glyoxylate recycling, the glyoxylate regeneration cycle (GRC), overlaps with the TCA and EMC cycles in type II methanotrophs and is considered as an additional route for glyoxylate regeneration [40,43]. Thus, the serine, EMC, and GRC pathways lead to mapping the derivatization of the secondary metabolites in type II methanotrophs. In nutrient-deficient conditions, an additional pathway to store energy in the form of polyhydroxybutyrate (PHB) granules is engaged [44], and is discussed in the following section.

## 3. Microbially Recovered Resources from Methanotrophic Bacteria and Their Biomedical Applications

**Figure 3:**
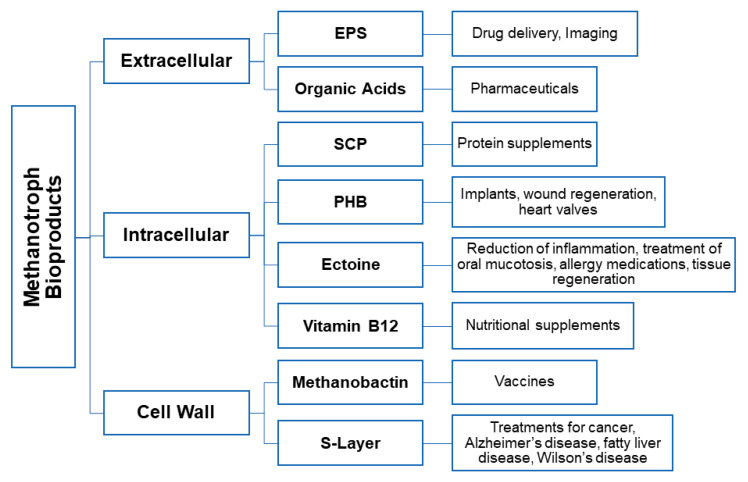
The diverse potential bioproducts synthesized by methanotrophs and their biomedical applications.

The unique metabolism of the methanotrophs enable them to both mitigate the greenhouse gas methane and remove harmful contaminants such as ammonia and nitrate found in water systems. Furthermore, they have the potential to produce valuable bioactive derivatives through methane uptake and phosphorylation pathways including single cell protein, biopolymer, S-layer, the copper binding protein methanobactin, methanol biogas, organic acids, ectoine, vitamin B_12_, and various enzyme catalysts. These products have significant value in diverse biomedical fields as they can be used as tools to aid in overcoming biomedical obstacles (Figure 3).

### 3.1. Exopolysaccharides

The extra-polymeric substance or exopolysaccharide (EPS) is a biocompatible, non-toxic, and decomposable high molecular weight carbohydrate-based polymer [45]. EPS is a primary component of biofilms in environments with low nutrient availability and/or high contamination to protect bacterial cells from environmental toxicity [46]. They are mainly formed by polysaccharide and protein integration, with low amounts of DNA and lipids being detected [46], and perform many roles as they ensure structural stability of the bacterial biofilm and act as a filter to allow the passage of certain nutrients while blocking the entrance of other molecules [47]. EPS production in methanotrophs has been found to be associated with high carbon-to-oxygen and -nitrogen ratios. Methane-rich environments such as soil interfaces are found to possess the thickest EPS biofilms [46]. The EPS is thought to play a role in carbon assimilation in different environments where nitrogen is depleted and type I methanotrophs are unable to fix nitrogen; EPS is produced through the RuMP pathway as a carbon reservoir [47]. On the other hand, type II methanotrophs have been known to occasionally produce the EPS polymer to catalyze nitrogen fixation by limiting oxygen penetration, which in turn causes oxygen depletion that triggers enzymatic activation [44]. Generally, in both type I and II methanotrophs, glycolysis is the main pathway for EPS synthesis as it metabolically overlaps with both the serine and RuMP pathways to provide nucleoside diphosphate saccharides that polymerize into EPS [48].

Microbial extracted EPS have better properties for industrial applications than its algal- and plant-based counterparts due to the replicability and sustainability of its production process and the higher quality polysaccharide polymer [47]. Additionally, the microbial EPS is of a stronger titer than the plant-based product. The safe biocompatible nature of EPS makes it an ideal candidate for many medicinal and pharmaceutical applications. An example of a microbially produced EPS is dextran, first used as a plasma expander to control bleeding in hypovolemic patients [49]. Moreover, bacterial alginates and other EPSs such as xanthan, pullulan, and bacterial cellulose are suitable in different applications, such drug encapsulation, dental casting material, treatment of acid reflux, and as scaffolds or wound dressings [50]. Moreover, hyaluronic acid produced by many bacterial species is used for many cosmetic purposes including skin regeneration, as well as in many operative procedures due to its ability to accelerate healing, and as a curative approach for arthritis [51]. Bacterial gellan is an EPS useful in the manufacturing of different medicinal drugs [52]. Similarly, EPS has seen application as additives to vaccine formulations and integration in devices for diagnostic imaging. Lastly, due to their physiological potency, EPS is often explored as a component for applications in immunoregulation and cancer treatments.

### 3.2. Polyhydroxyalkanoate

Polyhydroxyalkanoates (PHAs) are optically active microbial synthesized polyesters of hydroxy-acids; repeating monomers accumulated intracellularly as hydrophobic inclusion bodies ranging from 0.2–0.5 μm in diameter within the cytoplasm. These polymers are stored as an energy reserve material and can reach as much as 90% of the cell dry weight (CDW) in some species such as *Bacillus megaterium*. PHAs are accumulated under limiting conditions of phosphorus, nitrogen, and oxygen as well as excess carbon [53].

PHAs are non-toxic, biocompatible, isotactic and insoluble in water. PHAs tend to have a high polymerization rate, crystallinity and high molecular mass, which is a comparable property with regular plastics such as polypropylene (PP) [54]. PHAs have been classified into more than 150 different types based on the large number of different hydroxy-alkanoic acid monomeric structures with different side chain lengths. Therefore, they are divided into two main categories; short chain length (SCL) with 3-5 carbon molecules of hydroxy-acids monomers such as poly 3-hydroxybutyrate (PHB) and polyhydroxyvalerate (PHV) or medium chain length (MCL) 6-16 carbon molecules such as polyhydroxyoctanoate (PHO) [11]. The precursors of these PHA categories encompass the type of bacteria and the carbon substrate in the culture media; each category is characterized with different chemical and physical properties as well as designated industrial applications. For instance, the MCL PHAs have lower crystallinity and are more flexible than PHB or PHV [14,55], while SCL PHB is considered the most well studied PHA. Bacterial PHB has 55–80% crystallinity and mechanical properties similar to that of PP. However, it is less likely to break and is remarkably more durable than PP [56]. PHA can be produced using both Gram-negative and Gram-positive bacteria. However, it is noteworthy that most of the PHA production research have been focusing on Gram-negative bacteria even though Gram-positive bacteria, especially *Bacillus* spp., have long been found to produce large amounts of PHB. Moreover, Gram-negative microorganisms have a lipid bilayer that is not biocompatible and stimulate defense reaction by the body; hence, Gram-positive bacteria are better suited for biomedical applications.

PHB is synthesized by type II methanotrophs using the serine pathway in order to produce malyl-CoA, which is responsible for the initiation of the EMC cycle. PHB synthase is the terminal enzyme involved in the process of moderate final monomer formation. It is part of a group of enzymes encoded by the genes *phaA*, *phaB,* and *phaC*. An important group of PHA associated proteins, the so-called phasins, have been found to modulate the accumulation of the PHB polymer through regulation of the activation of *phaA*, *phaB*, and *phaC* expression. Methanotrophs have been found to produce high yields of PHB (up to 70% CDW) and have a vital role in the industrial accumulation of PHB [7,57].

PHA industrialization began in the 1990s where the mass production of this bioplastic was manufactured for multiple medicinal and non-medicinal purposes. While PHA has been offered as an answer to petroleum-based plastics, its high capital cost made it more suitable for biomedical and pharmaceutical applications [58]. Since PHA is an ecofriendly and biocompatible material, with an array of diverse physicochemical properties that can be modified to meet suitable strength and elasticity, it could be employed for a variety of applications ranging from hard to soft tissue engineering, implant manufacturing [58], tissue regeneration [7], vascular systems [59], heart valves [60,61], and bones and cartilage [16]. The integration of PHA polymers and copolymers within the human body help overcome some of the problems associated with using other materials including general fragility, danger of contamination, high chance of occlusions, or inducing an immunological response due to non-biocompatibility [60]. Hence, current applications of PHB and PHO have been employed as copolymers to create stents, bioactive tissue patches for heart and vascular systems, orthopedic pins, nerve guides, and repair devices, etc. [61,62,63,64,65]. Moreover, many important pharmaceutical applications include different form of drug carriers with the ability of controlled drug release [66].

### 3.3. Surface Layers

Surface layers (S-layer) are polymeric proteins that cover the outside of a microbial cell. They are of special importance as they can comprise ~15% of the total protein content of the cell [67]. These crystalline structures are characterized with an amorphous layout that mainly consists of hydrophobic molecules, acidic amino acids, and lysine [64]. This nanoscale lattice matrix is characterized with identical pore sizes, allowing for self-assembly in a variety of environments [65]. The negatively charged two-dimensional crystalline protein or glycoprotein lattice embodies the complex network of proteins involved in vital cellular functions such as synthesis, excretion, and the layout of membrane bound proteins [68]. While the specific role of S-layer has been controversial, some studies illustrate that the S-layer might be serving a different function for different bacterial groups, as part of an adaptation evolutionary mechanism in a diversity of eco-niches [68]. For example, the S-layer serves as a protective layer against high osmotic pressure where the porous membrane has the ability to counteract the external pressure in halophilic bacteria. Its ability to withstand high temperature and mechanical stress has been linked to maintenance of cell shape and structural integrity for archaeal groups [46], and the S-layer provides gram positive bacteria with a periplasmic space [69]. The S-layer guarantees the presence of binding sites for different exoenzymes, for instance providing linkage sites for thermostable proteases enabling tolerance to thermophilic environments, or the ability to incorporate enzymes like exo-amylase in *Bacillus stearothermophilus* [45]. The S-layer can also provide cellular adhesion capabilities [47], act as a selective semipermeable membrane to protect from lyase activity, and prevent parasite and toxin penetration, while at the same time modulating the passage of essential molecules and some important enzymes, and aid in retention of organic substrates and metal compounds bound to cellular membrane [65]. Lastly, in particular to methanotrophs, S-layer proteins have been linked with conveying copper ions to pMMO, which aids in maintenance of the equilibrium in regard of copper concentrations [49]. The prevalence of copper-binding proteins CorA and MopE, and diheme periplasmic cytochrome C peroxidase CorB/Mca was detected in *Methylomicrobium album* BG8, *Methylotuvimicrobium alcaliphilum* 20Z, and *Methylococcus capsulatus* (Bath) [70]. This protein combination corresponds to an iron chelating compound, methanobactin; methanotrophs that do not possess S-layers do not express CorA nor MopE polypeptide pairs [70]. The surface structure of aerobic methanotrophs is uniquely different since it permits multifunctionality which gives a communicative advantage between these microorganisms and their surroundings. However, it is important to unravel the genes responsible for the protein structure of S-layers in aerobic methanotrophs in order to understand the means of interaction between methanobactin and cell wall machineries that are able to make S-layers.

Slightly over four hundred different species of prokarya have been identified as possessing genes responsible for expression of S-layer [71]. It is noteworthy that the S-layer has little to no structural similarities between different taxonomical groups [65]. Furthermore, short sequences of post-translational mRNA are usually responsible for transcription of a single protein that makes up the S-layer crystalline matrix. The promoter’s sequences responsible for translation of S protein have dented long end 5′ untranslated region (UTR) which protects the protein from RNase dissimilative activity. S-proteins are characterized with a longer half-life, which is why it is stringently modulated to be synthesized in the stationary phase rather than the log phase that is achieved through regulatory repressive gene sequences to stop the translation of the S protein in the growth phase namely, *splA* in *Thermus thermophilus*. Hence, some species possess different genes responsible for each phase of cell synthesis [31].

In Gram-positive bacteria, S-layers are most commonly attached to the peptidoglycan layer through the SLH (S-layer homology) motif. On the other hand, in Gram-negative bacteria, S-layers are attached to the outer membrane lipid bilayer by ionically binding to the lipopolysaccharide or covalently joining N-terminal end to the S-layer and hydrophobic interactions as it can be joined with lipids via van der Waals forces. Accordingly, most methanotrophic bacteria such as *Methylococcus*, *Methylothermus*, and *Methylomicrobium* have an encapsulating S-layer with varying structure of the S-layer between methanotrophs [67]. Most methanotrophs S-layer form planar (p2, p4) symmetry, cup-shaped, or conical arrangements having hexagonal (p6) symmetry.

**Figure 4:**
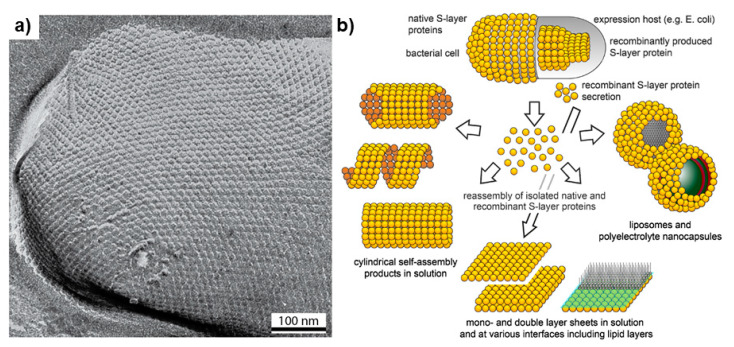
TEM micrograph (**a**) of an S-layer carrying *Methanocorpusculum sinense* cell and (**b**) the potential applications of reassembled of S-layer particles in solution, air water, and at surfaces (Adapted with permission from Pum et al. *Int. J. Mol. Sci*. **2013**, *14*, 2484–2501 [78]).

S-layers are highly organized matrices packed with proteins and glycoproteins; they have been found to be useful for many applications in biotechnology and nanomedicine (Figure 4) [64]. The S-layer differs within different bacterial species each with unique network of antigenic proteins [69]. Furthermore, S-layers possess good thermomechanical stability and the ability to capture different bioactive molecules [68]. In addition, S-layers are surrounded with a set of functional groups including protein biomarkers that have a role in preventing cellular damage by reducing oxidative stress, which are useful in different target therapy technologies [67]. Therefore, these crystalline formations show great promise in gene therapy and as drug nanocarriers. However, in some pathogenic microorganisms the S-layer can reflect virulence and potency. An example of this is *Bacillus anthracis* where the surface layer of this pathogen is responsible for causing anthrax. Therefore, S-layers are employed in vaccine development either as an attenuated pathogen, adjuvant, a hapten added to an immunization formula, or as vaccine carrier [72]. It has been found that S-layer containing vaccines to be a treatment for different hypersensitivity disorders such as type I allergy [73]. In addition, S-layers form highly stable ultrafiltration membranes that have uniform isoporous lattice with desirable mechanochemical stability that is formed by inter and intramolecular interactions which is useful for retaining important biological molecules and in the formation of lipid membranes [65]. S-layers have also been studied for creating immobilization matrices that can be used as a diagnostic tool to identify various diseases such as type I allergy [74,75]. The external layer contain a set of fusion proteins such as rSbpA, STII, and Cys, which are used in synthesizing gold nanoparticles [76]. Furthermore, some studies have demonstrated that S-layers belonging to different *Lactobacillus* strains have a role in protecting the human intestine as they form a protective coating that binds to the lining of the gut and creates a barrier in order to avert the attachment of many opportunistic pathogens like *E. coli* [77].

### 3.4. Methanobactin

Methanobactins (Mbns) are peptide chalkophores that bind copper, which is needed for cell synthesis [79]. They were originally characterized in the methanotrophic bacterial species *Methylococcus capsulatus* and were found to be required for the functioning of methane oxidizing pMMO [68], which is transported outside of the cell to trap and bind to Cu(I) ions [80]. This chelating effect also reduces Cu(II), which is toxic to the bacterial cell, and to Cu(I) in copper-deficient environments [81,82]. The Mbn-Cu(I) complex then enters the cell through active transport to convey copper for important metabolic functions in methanotrophic bacteria [83]. Mbns exhibit significant sequence variability between bacterial strains [84], and use a modified rRNA to regulate its translation [85].

Methanobactin acts as a chelating agent with the ability to bind to copper, mercury and gold particles [86]. This is especially important as the ability of reducing gold has successfully generated gold nanoparticles that have many biomedical applications. These applications include antimicrobial activity, incorporation in fuel cells, photo-thermal and dynamic therapy, cancer treatment, act as an epitope, and tether to immunogenic molecules that have the ability to attach to cancer biomarkers (antigens), and in the development of many medically advanced technologies such as plasmonic biosensors, for visualization, and bioimaging [80]. Mbns also show great potential in the treatment of copper-associated illnesses such as Wilson disease (WD), Alzheimer disease (AD), fatty liver disease, and BRAF-positive cancers by preventing copper buildup in the liver and consequently other body tissues, and thereby preventing permanent liver and neurological tissue damage [83].

### 3.5. Antibacterial Proteins

*Methylocystis minimus* and *Methylobacter luteus* have been found to produce a thermostable protein capable of killing pathogenic bacteria, which is currently under investigation for possible application as an antibiotic [13]. The bacteria encode genes that produce peptidase enzymes, which was found to function as bacteriocin [87,88].

### 3.6. Single Cell Protein (SCP)

Single cell protein (SCP) is a protein derived from microbial cells that feed on a range of organic carbon sources. Many microorganisms, including algae, blue-green fungi, and bacteria, which can make up to 80% CDW [18]. The current vernacular usage of SCP arose in 1966 following a substitution of an older term microbial proteins, which was used to describe dried microbial cells serving as an ingredient or a substitute for protein-rich foods [64]. In view of an insufficient world food supply, the use of biomass produced by industrially scaled reactors could in theory provide a resource for SCP recovery [89]. SCP is of great nutritional value because of its high protein, vitamins, and lipid and essential amino acid content [90]. Methanotrophs are a well-known source for SCP production where the proteinaceous substance within methanotrophic bacteria is estimated to be 60–65% [89].

SCP offers a source of vitamins, amino acids, minerals, crude fibers, etc., which are important for healthy eyes and skin [76]. It is used as a protein supplement for undernourished children as it is added to improve the nutritional value of many consumed products as well as for athletes as they consume to derive energy [91]. Furthermore, it is also used for animal nourishment including pigs, cattle, and poultry [92]. Currently, Spirulina tablets are prescribed as dietary supplement as it lowers blood sugar levels in diabetic patients due to the presence of gamma-linolenic acid and prevents the accumulation of cholesterol in human body [93].

### 3.7. Ectoine

**Figure 5:**
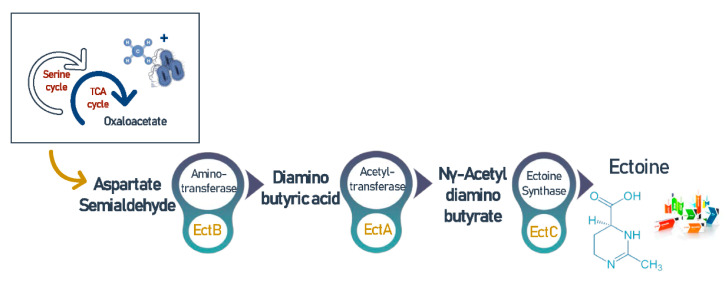
The cascade of reactions involved in the biochemical synthesis of ectoine.

Ectoine (C_6_H_10_N_2_O_2_) and its derivative hydroxyectoine (C_6_H_10_N_2_O_3_) are high value compatible solutes [94], and are secreted intracellularly by microbial cells in order to overcome an environment’s hypersalinity and balance the osmotic pressure [64]. It is noteworthy that this osmoregulatory can also be excreted to the extracellular environment as a response to hyposalinity [95]. Furthermore, ectoine synthesis was found to be associated with many amino acids, lipids, and proteins, which ensure their structural stability [31]. Thus, this cyclic imino acid has many medical and biotechnological applications and considered an expensive ingredient that currently retails for around $1000/kg [96]. Interestingly, in halophilic environments, methanotrophic bacteria synthesize ectoine by the conversion of oxaloacetate to aspartate followed by activation of a cascade reaction catalyzed by diaminobutyric acid (DABA) aminotransferase (EctB), DABA acetyltransferase (EctA), and ectoine synthase (EctC) as shown in Figure 5 [97]. This process is regulated by the MarR-like transcriptional regulator EctR1 that is usually found in conjunction with *ectABC* operon [94].

An important function for ectoine and hydroxyectoine is stabilizing and protecting nucleic acids and proteins from mutations. It is a key contributor to the prevention and treatment of many illnesses that are linked to protein and nucleic acid conformation such as Alzheimer’s disease (AD), Machado–Joseph disease (MJD), and spongiform encephalopathies that occur due to amyloid deformation [98]. Therefore, ectoine is important in mediating the hinderance of protein aggregation and chain elongation in these illnesses while reducing cytotoxicity [99,100]. Moreover, ectoine has an anti-inflammatory and hydrating nature which can prevent hostile immune responses [96]. This is achieved through reducing neutrophils activity and stabilizing epithelial cells through creating a barrier and obstructing inflammatory cell signaling initiation in the lungs, thus preventing diseases like oral mucositis (chemotherapy induced in cancer patients) and in the airways like Allergic Rhinitis (AR) and Rhinosinusitis (ARS). These hypersensitivity-related conditions are responsible for airway damage due to the increased oxidative stress caused by polluting nanoparticles in the air [101]. Topical application of ectoine solute as nasal or oral sprays have been shown to limit these conditions and to be effective as an allergy medication in order to prevent irritation [102]. Similarly, ectoine-based eye drops hydrating potential exhibit an analogous effect in controlling and treating eye inflammation which can be caused by many medically associated conditions causing eye dryness or irritation [103]. Ectoine is also employed and integrated in many skin care product formulations as it can protect skin from harmful UV-A radiation (due to its DNA stabilizing capability) while soothing skin irritations through a barrier to trap moisture and protect skin from damage [104]. Lastly, ectoine has an important role in the regeneration of body tissue and healing ulcers, treating conditions, such as vascular leak syndrome (VLS) and neurodermatitis [105].

### 3.8. Carotenoids

Carotenoids occur as a group of pigmented biomolecules that help in photosynthesis in a wide range of organisms such as plants, fungi, and bacteria. Carotenoids are divided into two classes: carotenes and xanthophylls. The former are biological precursors for the formation of vitamin A1 and retinol while the latter provide protection against excess absorbed light energy, therefore acting as a shield for organisms against photo-oxidative stress [106].

Carotenoids play an important role in biomedicine due to their antioxidant activity and oxygen reducing ability which endow a protective function to several organs such as the heart and pancreas. For instance, the presence of lycopene has been correlated with heart health and the prevention of cervical intraepithelial neoplasia [107] and myocardial infarction [108]. Furthermore, carotenoids has been found to regulate gene expression which in turn affects cell growth and overall immunity [109]. Cohort studies have demonstrated an association between high carotenoid intake and a reduced occurrence of breast, cervical, ovarian, colorectal cancers, cardiovascular, and eye diseases [110]. The *Methylomonas* genus of methanotrophic bacteria has been found to produce one form of xanthophylls, namely lycopene. Lycopene consists of a 40 carbon chain and is present in lipophilic mediums such as membranes and lipoproteins commonly found in type I methanotrophic bacteria [111]. This xanthophyll derivative is a well-known antioxidant that protects the cell against reactive oxygen species (ROS) and oxidative damage and is often associated with various chronic illnesses, including cancer and cardiovascular diseases. Likewise, studies have shown that a drop in lycopene levels in blood serum as one ages has been considered to have a role in the incidence of prostatic, cervical, and pancreatic cancers [110]. Furthermore, lycopene plays a pivotal role in protecting against coronary atherosclerosis and myocardial infraction by being a part of low-density lipoproteins (LDL) and promoting immunity against free radicals damage [112]. Due to the aforementioned functional role of carotenoids, they have been employed in the drug industry and cosmetics, since they are biological precursors for vitamin A production, an fundamental nutrient in biological functions comprising vision, reproduction, and immunity [113]. As well, carotenoids are used as additives in the form of lutein and astaxanthin which exhibit anti-inflammatory and neuroprotective effect [114]. The administration of a mixture of lutein, β-cryptoxanthin, lycopene, zeaxanthin, and fucoxanthin have been found to inhibit function of the p16 and p73 oncogenes [115]. Different types of carotenoids are used in treatment of several illnesses such as diabetes, Parkinson’s, Alzheimer’s (Table 1).

## 4. Outlook and Practical Implications

Many microbial organisms inhabiting wastewater treatment plants have the potential to be used in biomedical applications including genera of *Methanotrophs*, *Nitrosomonas*, *Pseudomonas*, and *Bacillus*. In addition to important bacterial isolates found in WWTPs, there are also algal species such as *Chlorella*, *Dunaliella*, *Sargassum*, and *Enteromorpha* that are able to produce extracts like isoflavones, pigments, phenolics, carotenoids, polysaccharides, vitamins, and minerals. Advancements in the extraction and purification of bioactive compounds has led to its employment as a main ingredient in developing drug delivery systems, hormonal therapy, and biocompatible medical materials. [118]. In addition to the variety of applications in pharmaceutical and biomedicines of these extracts, WWTPs are also rich with bacterial strains such as *E. coli* that possess the ability to produce therapeutic recombinant proteins using bacterial expression systems [119]. Consequently, many previously untreatable disorders have been treated using genetic engineering of prokaryotic expression machinery such as diabetes, obesity, sexual dysfunction, and psychological disorders and have been guided by the biosynthesis and purification of many bioactive compounds such as insulin, neurotransmitters [120] and synthetic hormones [121]. Moreover, advancements in the dermatological industry have been made due to the emergence of biological ingredients such as Kojic acid, retinols, nitric oxide, penicillin and terbinafine, which have proven their efficacy in the treatment of many skin related conditions such as hypo/hyperpigmentation, psoriasis, injuries, bacterial and fungal infections, respectively. The antimicrobial, anti-tanning, texture enhancing, anti-wrinkling, skin sensitizers, soothing, repairing, and regenerating properties of these molecules have contributed to their extensive employment in skin care products in the last two decades [122,123]. Similar to pharmaceutical and dermatological industries, biotechnological advancements in the field of biomedicines have also been reported. Many bacterial and algal species have been used to produce different high-value nanoparticles that have applications in imaging, diagnostics, as antimicrobial agents and novel nano-based technologies such as surgical nanobots used in non-invasive procedures, prosthetics and tissue engineering [124]. Here, we reviewed an example of a WWTP isolate, methanotrophic bacteria and their bioproducts, for potential applications biomedicine. Biomolecules purified from methanotrophs have demonstrated application in the manufacturing of implants, scaffolds, drugs, dental casting, sutures for surgical procedures, prosthetic, supplements, vaccines, drug additives, and castings. In addition to their use in diagnostics, these biomolecules are also useful in the treatment of conditions such as, arthritis, cancer, autoimmune diseases, Wilson disease, Alzheimer’s disease, fatty liver disease, and BRAF-positive cancers, a wide variety of infections, oxidative damage of many organs, vascular leak syndrome, neurodermatitis, bleeding, and wounds, as well as the prevention of cancer, liver cirrhosis, neurological tissue damage, skin conditions, diabetes, coronary heart disease, and osteoporosis.

## 5. Conclusions

There is an interplay between advances in biomedicine and biotechnology and other fields of study including microbiology, agriculture, and engineering. Taking advantage of available tools and assets that exist in industrial systems is an opportune approach for next-generation solutions. The relationship between the biomedical industry and water treatment facilities may appear tenuous at first glance; however, they are intercorrelated, and the exploration of unexploited resources such as methanotrophic bacteria in WWTPs can lead to novel biomedical advances. There is both great value and significant potential for biotechnological and biomedical applications stemming from bioproducts produced by methanotrophic bacteria found in WWTPs. While this review covered some examples of products recovered from methanotrophic bacteria, there are many other potentially beneficial products that can recovered from wastewater facilities that have important applications in biomedicine and biotechnology.

## Figures and Tables

**Table 1:** The role of different carotenoids in disease prevention.

Carotenoid	Disease(s)	Mode of Action	Reference(s)
α-carotenes, β-carotene	Diabetes, dyslipidemia, and hyperhomocysteinemia	Reduction fatty acid radicles and reactive oxygen species play vital role in increasing the GR, GPx, and other hormones leading to diseases Malaria, HSV-1, keratitis, endophthalmitis, conjunctivitis	[108,109,116]
Astaxanthin, lutein, β-cryptoxanthin, lycopene	Heart disease	Oxidizing LDL and decreasing HDL	[117]
Lutein and zeaxanthin	Macular degeneration and cataract	Filters for blue light from screens Free radical scavenger in the retina	[106]
Astaxanthin, β-carotene and lycopene	Alzheimer’s, Huntington’s, Parkinson’s, and amyotrophic lateral sclerosis (ALS)	Anti-apoptosis, reduction in cerebral infarction in brain tissue, lowers ischemia by induced apoptosis, reduction of glutamate release and reduce free radical damage Lycopene increases blood–brain barrier permeability, and it reduces when certain diseases occur Enhance calcium ion transport to the brain; improve brain signaling	[107]
β-cryptoxanthin	Osteoporosis	Enhanced the activity of alkaline phosphatase and calcium content in metaphyseal tissue and cortical bone	[114]
β-cryptoxanthin, lycopene and β-carotene	Ovarian, breast, prostate, cervical, and liver cancer	Downregulation of cyclin D1, cyclin D2, CDK4 and CDK6expression; it also upregulates GADD45 α, which inhibits the entry of cell into S phase Suppressing the NF-κb signaling pathway anti angiogenic activity	[110]
Astaxanthin, β-carotene, lycopene, lutein, and β-cryptoxanthin	Liver damage	Protection for cells, lipids, and membrane proteins towards oxidative damage	[114]
β-carotene, canthaxanthin and lycopene	Melanoma	Decrease UV-light mediated damage	[117]

## Data Availability

Not Applicable.
